# Evaluation of Adverse Effects of Resorbable Hyaluronic Acid Fillers: Determination of Macrophage Responses

**DOI:** 10.3390/ijms23137275

**Published:** 2022-06-30

**Authors:** Wim H. De Jong, Danyel Jennen, Peter H. J. Keizers, Hennie M. Hodemaekers, Jolanda P. Vermeulen, Frank Bakker, Paul Schwillens, Marcel van Herwijnen, Marlon Jetten, Jos C. S. Kleinjans, Robert E. Geertsma, Rob J. Vandebriel

**Affiliations:** 1 Centre for Health Protection, National Institute for Public Health and the Environment (RIVM), 3720 BA Bilthoven, The Netherlands; peter.keizers@rivm.nl (P.H.J.K.); hennie.hodemaekers@rivm.nl (H.M.H.); jolanda.vermeulen@rivm.nl (J.P.V.); frank.bakker@rivm.nl (F.B.); paul.schwillens@rivm.nl (P.S.); robert.geertsma@rivm.nl (R.E.G.); rob.vandebriel@rivm.nl (R.J.V.); 2 Department of Toxicogenomics, GROW School for Oncology and Reproduction, Maastricht University, 6200 MD Maastricht, The Netherlands; danyel.jennen@maastrichtuniversity.nl (D.J.); m.vanherwijnen@maastrichtuniversity.nl (M.v.H.); marlon.jetten@maastrichtuniversity.nl (M.J.); j.kleinjans@maastrichtuniversity.nl (J.C.S.K.)

**Keywords:** resorbable fillers, hyaluronic acid, adverse effects, macrophage responses

## Abstract

Resorbable tissue fillers for aesthetic purposes can induce severe complications including product migration, late swelling, and inflammatory reactions. The relation between product characteristics and adverse effects is not well understood. We hypothesized that the degree of cross-linking hyaluronic acid (HA) fillers was associated with the occurrence of adverse effects. Five experimental HA preparations similar to HA fillers were synthesized with an increasing degree of cross-linking. Furthermore, a series of commercial fillers (Perfectha^®^) was obtained that differ in degradation time based on the size of their particulate HA components. Cytotoxic responses and cytokine production by human THP-1-derived macrophages exposed to extracts of the evaluated resorbable HA fillers were absent to minimal. Gene expression analysis of the HA-exposed macrophages revealed the responses related to cell cycle control and immune reactivity. Our results could not confirm the hypothesis that the level of cross-linking in our experimental HA fillers or the particulate size of commercial HA fillers is related to the induced biological responses. However, the evaluation of cytokine induction and gene expression in macrophages after biomaterial exposure presents promising opportunities for the development of methods to identify cellular processes that may be predictive for biomaterial-induced responses in patients.

## 1. Introduction

Resorbable soft tissue fillers are currently popular for use in cosmetic treatment, as shown by an increase in use in the Netherlands between 2016 and 2019 estimated to be up to 160,000 in 2019 [1]. In addition to Botox, a botulinum neurotoxin, exerting a temporary effect on facial contour, resorbable fillers such as polylactic acid, polycaprolacton, hydroxylapatite, or hyaluronic acid (HA) show a more prolonged effect period. The most popular types of fillers contain HA which is composed of a linear polysaccharide naturally present in the body [2].

Injectable tissue fillers applied for aesthetic purposes may lead to severe complications including product migration, unexpected late swelling and inflammatory reactions such as granulomas [3,4,5]. Unfortunately, the cause of the failure, i.e., being treatment- or product-related, is often not specified [3]. The use of non-resorbable fillers, which has been recognized as problematic especially in the longer term [4], has been greatly reduced. Since January 2015, non-resorbable fillers are only allowed for reconstructive purposes, and are therefore banned for cosmetic use in The Netherlands [6]. An increasing number of resorbable fillers are becoming available, among which some may also unfortunately show long-lasting adverse effects somewhat similar to non-resorbable fillers. For example, both HA and calcium hydroxylapatite may induce nodules at various times during and after injection, with or without inflammation [7,8]. In contrast, an overview indicated limited adverse reactions after using calcium hydroxylapatite for unapproved indications [9]. In an evaluation of more than 2000 patients injected with HA, poly-L-lactic acid or calcium hydroxylapatite, the most common complication was nodule or granuloma formation with a relatively low incidence [10]. Even with a relatively low incidence, however, given the number of people treated with these fillers, a rather high number of patients will suffer complications.

In order to minimize the frequency and number of treatments, a more prolonged effect of injected cosmetic fillers is preferable. On the other hand, in view of the possibility for adverse responses, a prolonged presence may trigger more intense rejection responses and thus adverse reactions. The level of cross-linking and HA concentration are some of the features determining degradation and thus local persistence [11]. Although HA is a naturally occurring molecule present in the body, it is the manipulation of the molecule (e.g., cross-linking, particulate physical form) that results in recognizing the filler as a foreign body [12]. Thus, in addition to the initial inflammatory response by neutrophilic granulocytes immediately after injection, additionally, during prolonged presence, a foreign body response might develop. The extent and severity of such a foreign body response ultimately results in an adverse effect. Indeed, the biocompatibility of HA-based materials was reported to decrease with the number of modifications to which this polysaccharide was subjected [13]. The product Hyacorp was removed from the market in 2012 due to a relatively high number of adverse effects. Keizers et al. [14] showed that a high modification grade and cross-linking grade might be correlated with a higher risk of adverse effects. We hypothesized that manipulation by cross-linking HA chains may result in driving the macrophage immune responses into either a more fibrotic or a more inflammatory orientated response. The hypothesis is evaluated in this study by in vitro macrophage stimulation with extracts from various HA filler preparations. However, it should be realized that in addition to product characteristics, the injection procedure on its own could also lead to adverse events [15].

Currently, fillers composed of HA have become one of the most popular materials used for soft tissue contouring [16]. A large number of products are available that can differ in HA sources (primarily rooster comb or bacterial), cross-linkage (agent and degree), HA concentration, hardness, cohesivity, consistency, and longevity of the resulting correction [16]. For a number of these products, advantages and disadvantages were reviewed [16,17,18], whereas an overview of approved HA fillers is presented on the FDA website [19]. Currently, FDA-approved dermal fillers containing an HA comprise up to 23 different products. However, due to the manipulation and formulation of these HA-based products, it is likely that adverse effects will also occur for these resorbable fillers. Transient local swelling but also more serious swelling has been reported [20,21]. An advantage of HA fillers is that potential excessive reactions and/or swelling can be treated with hyaluronidase, resulting in a decrease in the swelling [22,23]. Incidentally, even late occurring adverse events (e.g., delayed onset nodules, granulomatous foreign body reaction, severe edema) have been reported [24,25,26]. However, the relation between product characteristics and adverse effects is not well understood. Thus, more knowledge is needed on the type of potential complications and the mechanism of the induction of these adverse responses.

The safety evaluation of soft tissue fillers should be risk-based and follow the principles used for medical devices. This depends on the type, use, and time of contact of the medical device with a patient, and the specific types of safety testing depend on the identified potential risks for the patient [27]. In this context, there are still biological processes and reactions against implanted biomaterials that are far from understood. In addition, the prediction of long-term effects is not really possible. In order to make this possible, more knowledge needs to be generated on the mechanism of the interaction of the biomaterial with tissues and cells. Neutrophils followed by monocytes differentiating into macrophages are the first cells to respond to implanted medical devices initiating an acute inflammatory response that eventually results in the so-called macrophage directed foreign body response, the end-stage response of the inflammatory and wound healing reactions after the implantation of a medical device, prosthesis, or biomaterial [28,29]. Material characteristics including the source of the biomaterial, e.g., tissue-derived or synthetically manufactured, can affect the polarization of macrophages [30]. By identifying the macrophage response both at the cellular and molecular levels, insight is obtained into the processes involved in the tissue response to an implanted material. The aim of this study was to identify potential cellular and/or molecular processes that may be predictive for HA-based filler material responses in patients.

Grotenhuis et al. [31] previously reported on a human macrophage culture model that could be used to evaluate macrophage responses to biomaterials. In this study, we used the macrophages derived from a human monocyte cell line (THP-1) to evaluate cytokine induction by various HA fillers. We hypothesized that late adverse reactions after the injection of resorbable HA tissue fillers could be related to the degree of cross-linking of HA chains in injectable tissue fillers. This will have direct influence on the dynamics and kinetics of the degradation and absorption process of the injected product. Depending on the degree of cross-linking, further chemical and/or enzymatic breakdown may occur, or the fragments may locally activate macrophages, which then induce the inflammation/granuloma formation. A series of HA samples with different levels of cross-linking were prepared, and their reactivity was compared to a series of commercial HA fillers (Perfectha^®^) with increasing sizes of the composing particulates resulting in a prolonged presence at the injection site.

## 2. Results

### 2.1. Effect of HA Fillers on Cell Viability and Cytokine Production

In preliminary experiments with two commonly used murine cell lines (macrophage cell line RAW264.7 and fibroblast cell line L929), no cytotoxic effects were noted for the experimental RIVM preparations and the Perfectha^®^ fillers, whereas the non-resorbable filler Bio-Alcamid^®^ and the positive control DMSO clearly induced cytotoxicity as indicated by a reduced metabolic activity and decreased membrane integrity (see Supplementary Figures S1 and S2).

**Figure 1:**
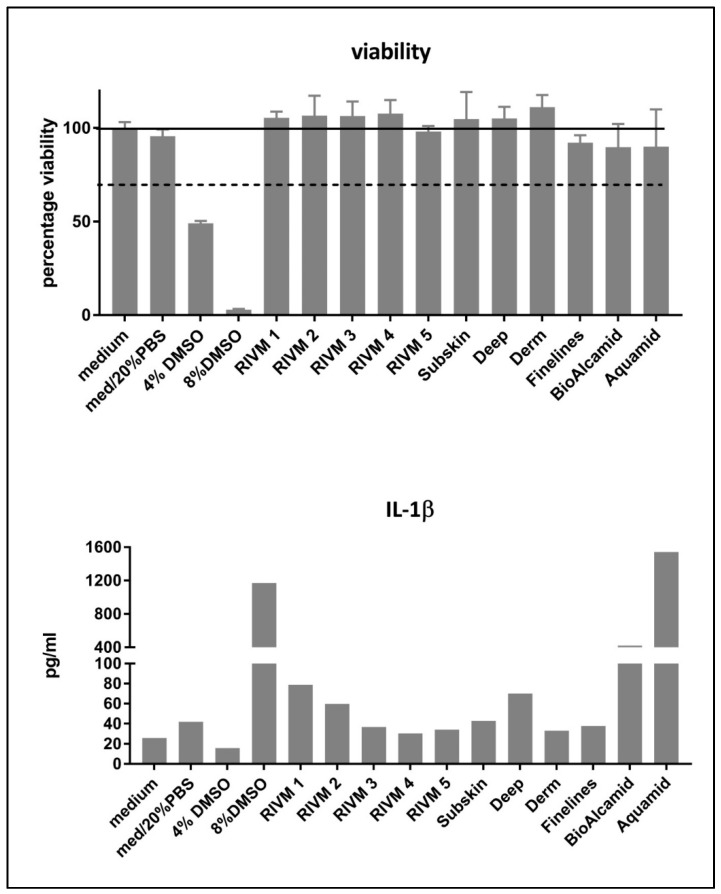
Effect of RIVM and Perfectha^®^ (Subskin, Deep, Derm, Finelines) HA fillers, and DMSO on cell viability. Results are presented as mean ± SD of three independent experiments while in each experiment, incubations were performed in four-fold. Top: Viability of THP-1-derived macrophages after 24 h exposure to various HA filler extracts. The 70% cell viability is indicated as the level for the indication of cytotoxicity (ISO 10993-5:2009). Bottom: IL-1β production by THP-1-derived macrophages after exposure to various HA filler extracts. RIVM preparations #1–#5 have an increase in cross-linking of HA chains by BDDE from #1 to #5. Perfectha^®^ fillers have a decrease in particle size from Perfectha^®^ SubSkin to Perfectha^®^ FineLines.

The cell viability and induction of IL-1β (as a marker for M1 macrophages) by M0 macrophages derived from monocytic THP-1 cells after exposure to the HA fillers is presented in Figure 1. The HA preparations and fillers did not induce cytotoxicity in the THP-1-derived M0 macrophages. In contrast to the preliminary experiments with murine cells, the THP-1-derived M0 macrophages did not show a cytotoxic response to the exposure of extracts from non-resorbable fillers Bio-Alcamid^®^ and Aquamid^®^. Only DMSO, included as positive control, induced a reduction in cell viability of the THP-1-derived M0 macrophages. The M0 macrophages exposed to DMSO showed an increase in IL-1β production while cell survival was low. The M0 macrophages incubated with various fillers showed a small increase in IL-1β production while there was no effect on cell viability (Figure 1). The non-resorbable fillers Bio Alcamid^®^ and Aquamid^®^ induced high levels of IL-1β production.

**Figure 2:**
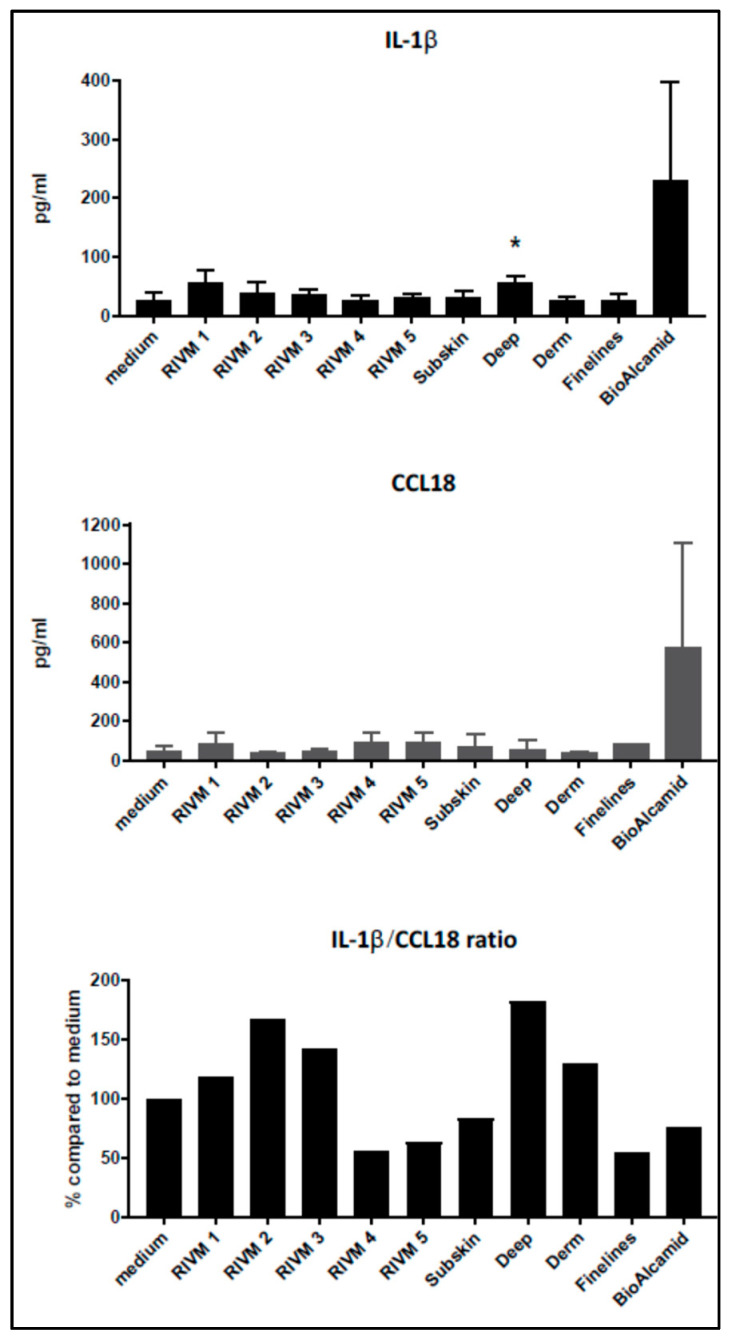
Effect of RIVM and Perfectha^®^ (Subskin, Deep, Derm, Finelines) HA fillers and Bio-Alcamid^®^ non-resorbable filler on cytokine production by THP-1 cells. Results are presented as mean ± SD of three independent experiments while in each experiment, incubations were performed in four-fold. IL-1β and CCL18 induction in M0 macrophages derived from monocytic THP-1 cells after exposure to extracts of RIVM preparations and commercial Perfectha^®^ HA fillers and of the non-resorbable filler Bio-Alcamid^®^. RIVM preparations #1–#5 have an increase in cross-linking of HA chains by BDDE from #1 to #5. Perfectha^®^ fillers have a decrease in particle size from Perfectha^®^ SubSkin to Perfectha^®^ FineLines. * *p* < 0.05. Student’s *t*-test.

Grotenhuis et al. [31] suggested to establish the ratio of M1–M2 markers as a means of assessing the pro-inflammatory versus pro-fibrotic response to various biomaterials. For this purpose, IL-1β and CCL18 were evaluated as M1 and M2 markers, respectively. The non-resorbable filler Bio-Alcamid^®^ strongly induced the production of both IL-1β and CCL18 (Figure 2; mean ± SD of three independent experiments), whereas the HA fillers only showed a rather small increase in cytokine production. No clear differences are observed in the cytokine levels upon treatment with the various resorbable fillers.

In line with this approach, we calculated the IL-1β (M1)–CCL-18 (M2) ratio (Figure 2; tissue culture medium control is set at 100%). From the fillers prepared at RIVM, the two with the highest cross-linking percentage showed the lowest IL-1β/CCL18 ratio, suggesting that they induced the highest pro-fibrotic response. The commercial fillers (Perfectha^®^) showed a gradual decrease in the IL-1β/CCL18 ratio in the order Deep, Derm, SubSkin, FineLines, suggesting a stronger pro-fibrotic response with a smaller filler size.

### 2.2. Effect of HA Fillers on Cell Activity Determined by Microarray Analysis

The cellular response of THP-1-derived M0 macrophages after exposure to the extracts of a range of dermal fillers was investigated by measuring the genome-wide gene-expression. Following data pre-processing, the number of probes used for identifying differentially expressed genes (DEGs) was 24,788 for M0 macrophages exposed to RIVM preparations and Bio-Alcamid^®^ extracts and 24,382 for M0 macrophages exposed to Perfectha^®^ filler extracts. The number of DEGs based on an absolute FC > 1.5, and an adjusted *p*-value < 0.05 for the in vitro exposed macrophages is shown in Table 1 and Table 2. Furthermore, for those comparisons, showing a low number of DEGs for an adjusted *p*-value < 0.05, a less stringent cut-off was also used, i.e., an adjusted *p*-value < 0.2.

Gene expression changes of *IL-1β* and *CCL18* after the exposure of the macrophages to the various HA-based fillers and Bio-Alcamid^®^ are shown in Table 3. For the non-resorbable filler Bio-Alcamid^®^ a significant up-regulation (adjusted *p*-value < 0.05) is observed for both *IL-1β* and *CCL18*, whereas for the resorbable fillers, only *CCL18* has an elevated gene expression (FC > 1.5 or log2 FC > 0.585). These results are in line with IL-1β and CCL18 production (Figure 1 and Figure 2). The expression changes of other cytokines as well as chemokines are available as Supplementary Table S1. Whereas many cytokines and chemokines hardly showed any expression for most of the exposures, the pro-fibrotic cytokine *CXCL8* (also known as *IL-8*) showed an elevated expression for most exposures, i.e., *CXCL8* is significantly up-regulated (adjusted *p*-value < 0.05) for Bio-Alcamid^®^ and all Perfectha^®^ fillers.

### 2.3. Pathway Analysis

**Figure 3:**
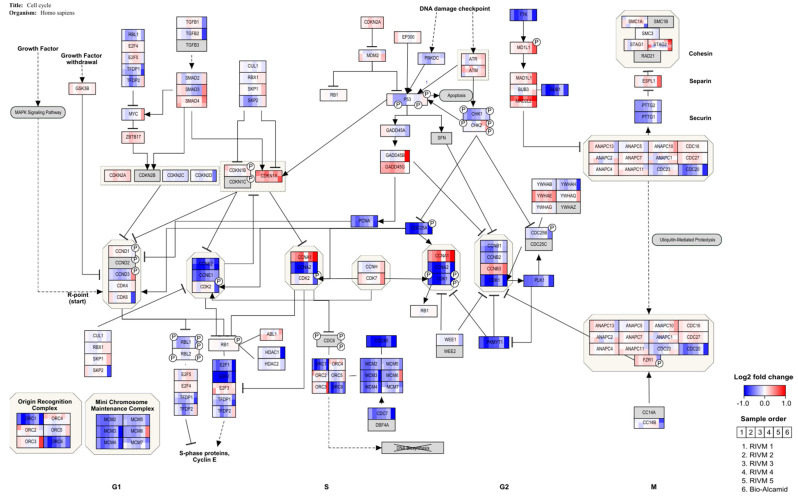
Visualization of gene expression changes for M0 macrophages derived from THP-1 cells exposed to RIVM preparations and Bio-Alcamid^®^ extracts on the cell cycle pathway. Cells were exposed for 24 h to extracts of the various HA fillers. At 24 h, the cells were harvested and RNA isolated for cDNA preparation and microarray gene analysis.

**Figure 4:**
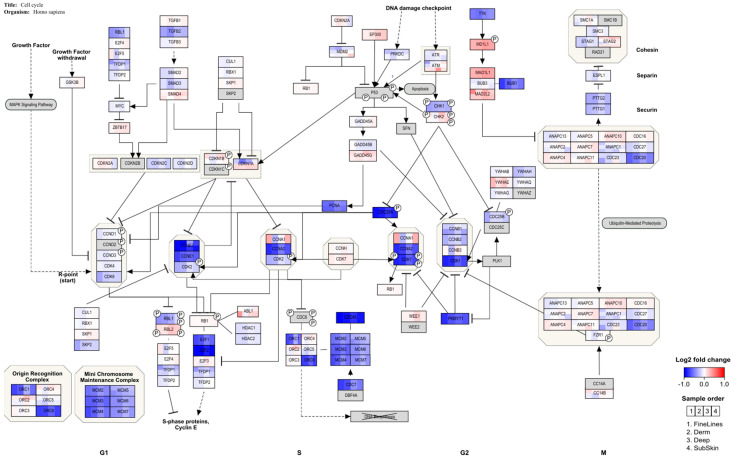
Visualization of the gene expression changes for M0 macrophages derived from THP-1 cells exposed to Perfectha^®^ filler extracts on the cell cycle pathway. Cells were exposed for 24 h to extracts of the various HA fillers. At 24 h, the cells were harvested and RNA isolated for cDNA preparation and microarray gene analysis.

An over-representation analysis in PathVisio was performed for the selected DEGs of the in vitro experiments. In Table 4, Table 5 and Table 6, the top significant pathways are shown for the different gene sets. A full overview of the pathway analysis results is available as Supplementary Table S2. Cut-off values, i.e., adjusted *p*-values, for DEG selection using moderated *t*-tests are indicated. No pathway analysis was conducted for RIVM 4 and 5 preparations as the number of DEGs was too low, even when applying the less stringent adjusted *p*-value < 0.2. Different pathways are highlighted related to immune response and cell cycle control. For the Bio-Alcamid^®^ filler extract, the highest scoring pathways were related to immune responses such as macrophage markers and cytokine signaling. For both RIVM preparations and the Perfectha^®^ filler extracts, the cell cycle pathways were altered. Most genes in the cell cycle pathway are down-regulated (Figure 3 and Figure 4) which is indicative of cell cycle arrest. Additionally, for the Bio-Alcamid^®^ filler, most of these cell cycle genes are down-regulated (Figure 3), despite the fact that the cell cycle-related pathways are not significantly altered. A full overview of the expression of the genes in the cell cycle pathway shown in Figure 3 and Figure 4 is available as Supplementary Table S3.

## 3. Discussion

This study investigated the various aspects of HA fillers including the determination of the cross-linking degree of the HA fillers, the in vitro cytotoxicity of the HA fillers, possible induction of a macrophage pro-inflammatory/pro-fibrotic response, and the effect of the HA fillers on gene expression in macrophages. A series of HA fillers with an increasing degree of BDDE cross-linking was synthesized to validate the method for the analysis of the cross-linking grade of HA-based filler commercial products [14]. Indeed, the RIVM 1–RIVM 5 preparations showed an increasing level of cross-linking of the HA chains. Our results show that both the experimentally synthesized RIVM preparations and Perfectha^®^ HA fillers did not elicit cytotoxic effects in a macrophage (RAW264.7) and fibroblast (L929) cell line (Supplementary Figures S1 and S2), and THP-1-derived M0 macrophages.

Cytokine induction in macrophages can be used to evaluate a possible macrophage response and their further maturation into M1 and/or M2 macrophages after exposure to biomaterials [31]. For the M1 phenotype associated with a more pro-inflammatory response the cytokines IL-1 β, IL-6, TNF-α, monocyte chemotactic protein (MCP)-3, and macrophage inflammatory protein (MIP)-1α can be evaluated, whereas for the M2 phenotype associated with a more fibrotic response, IL-1 receptor antagonist (IL-1RA), CCL18, regulated and normal T-cell expressed and secreted (RANTES), and macrophage-derived chemokine (MDC) can be used [31]. We used IL-1β production as a marker for M1 macrophage induction and CCL-18 production as a marker for M2 macrophage induction. Only DMSO included as positive control was clearly toxic for the cells (Figure 1). The induction of IL-1β and CCL18, as M1 and M2 markers, respectively, was evaluated in THP-1-derived macrophages. A high level of IL-1β production was observed for the DMSO positive control treatment. Additionally, the two non-resorbable fillers Bio-Alcamid^®^ and Aquamid^®^ induced a high IL-1β production at exposure levels that did not induce toxicity in M0 macrophages. In this respect, the M0 macrophages derived from the monocytic THP-1 cells might be less sensitive to the toxic effects of the non-resorbable filler Bio-Alcamid^®^ which was toxic for the murine RAW264.7 macrophage cell line. Bio-Alcamid^®^ induced both M1 (IL-1β) and M2 (CCL18) cytokines at high levels compared to the HA fillers evaluated (Figure 2). However, when we more closely considered the low levels of cytokine induction by the experimental and commercial HA fillers separately, some indications could be noted from the effects of the fillers prepared at RIVM. The two RIVM preparations with the highest cross-linking levels (RIVM 4 and 5) showed the lowest IL-1β/CCL18 ratio, suggesting that they might induce a pro-fibrotic response. The commercial fillers (Perfectha^®^) show a gradual decrease in the IL-1β/CCL18 ratio in the order of Deep, Derm, SubSkin, and FineLines, suggesting an increase in a more pro-fibrotic response with a smaller particle size in the fillers. This phenomenon can also be observed for the gene expressions of *CCL18* and *CXCL8*, where a higher up-regulation of these genes is evident for the fillers with a smaller particle size (Table 3 and Supplementary Table S1). An elevated expression of the pro-fibrotic cytokine *CXCL8* (also known as *IL-8*) upon exposure to Bio-Alcamid^®^ and all Perfectha fillers is in line with the elevated expression of M2-macrophage-associated chemokine *CCL18*, as *CCL18* induces an expression of *CXCL8* [32].

Our results indicate that the evaluation of the levels of M1 and M2 cytokines after the exposure of M0 macrophages may possibly be a model to evaluate the pro-inflammatory/pro-fibrotic characteristics of biomaterials including fillers. The production of the M1 cytokine IL-1β and the M2 cytokine CCL18 can be considered indicative for either a more inflammatory- or fibrotic-oriented local response, respectively. Such characteristics could link to complications such as nodules or granulomas, which are among the most commonly reported complications. It is clear that these data should be complemented with other M1 and M2 markers before a more definitive conclusion can be drawn, and the effects of these fillers on differentiated M1 and M2 macrophages should be investigated. Nonetheless, evaluating the levels of M1 and M2 cytokines upon the incubation of THP-1-derived M0 macrophages may possibly be a first step to a cellular model to evaluate the pro-inflammatory/pro-fibrotic characteristics of biomaterials including fillers.

Remarkably, the resorbable RIVM HA preparations and Perfectha^®^ HA fillers hardly induced gene pathways related to immune activity (e.g., cytokine signaling and macrophage markers), while they did induce gene pathways related to cell cycle control. On the other hand, the non-resorbable filler Bio-Alcamid^®^ did induce pathways related to immune activity, and only a few pathways related to cell cycle control. A clear explanation of why the resorbable HA fillers did not show activation of immune-related pathways, whereas the non-resorbable Bio-Alcamid^®^ filler did activate the immune-related pathways, is not obvious as both types of fillers showed no effect on the viability of the exposed cells; in other words, they were not cytotoxic for the exposed macrophages. However, Bio-Alcamid^®^ also showed a much higher induction of cytokines as presented in Figure 1 and Figure 2 when compared to the resorbable RIVM HA fillers. Therefore, it could be expected that the immune-related gene expression would be also increased. Bio-Alcamid^®^ is known to cause a relatively high number of more severe complications that require clinical intervention [33,34,35]. Thus, both the cytokine induction and altered gene expression could provide opportunities to develop methods for the preclinical safety evaluation of biomaterials. The in-house-made preparation RIVM 2 is the most reactive resorbable “filler” in our studies. This indicates that the gene expression response does not correlate with the number of cross-links. Bio-Alcamid^®^ is overall the most responsive filler in the macrophage cell model.

After HA filler exposure in the M0 macrophages derived from THP-1 cells, genes indicating cell cycle arrest at the G1 checkpoint are regulated, whereby *E2F* and several cyclins are down-regulated (Figure 3 and Figure 4). This is in line with a study in which THP-1 macrophages were challenged with retinoic acid, resulting in the down-regulation of *E2F* and *cyclin E* and resulting in cell cycle arrest [36]. In addition, this study also showed enhancement of the phagocytic activity of THP-1 cells. This would be in line with an adverse reaction to the dermal fillers. A clear inflammatory response, showing the up-regulation of several cytokines, is observed for a human full skin model after exposure to the same dermal fillers (Jennen et al., in preparation). This response can be considered to reflect an acute inflammatory response, as seen in patients injected with HA fillers [37].

Although the in vitro experiments may give a good indication of the biological processes that underlie the exposure of the cell models to HA fillers, a direct comparison with reactions in patients is needed to indicate the value of the in vitro macrophage cell model. However, a comparison with the patient samples (biopsies) of local adverse effects of HA fillers might be difficult to achieve. Procedures to obtain biopsies are invasive and can lead to scarring or other severe complications [38] and thus patients are reluctant to give consent for biopsies to be taken, especially coming from the facial area (Decates, personal communication). Additionally, adverse reactions can be clinically managed by applying hyaluronidase to the reaction site without causing (severe) complications [22,23]. The clinical relevance of our studies can be found in the predictive value of the evaluated test systems in the preclinical safety evaluation of biomaterials and/or medical devices. Both the fibroblast and macrophage responses, corresponding the formation of fibrotic tissue surrounding an implant and macrophages trying to phagocytize the implant as a foreign body, respectively, are normal biological responses to an implant that is not naturally present in a human body [28,29]. The extent of these reactions, i.e., severe fibrosis and/or capsule formation or a severe inflammation due to a massive foreign body response, determines whether these reactions should be seen as adverse.

In conclusion, our results could not confirm the hypothesis that the level of cross-linking in the experimental HA preparations or the particulate size in the commercial HA fillers is related to the biological (cytotoxic or immune) responses induced by the HA fillers. However, our results do present evidence that in vitro models either composed of a macrophage cell line or macrophages differentiated from a monocytic cell line may be used for the identification of immune pathways more prone for either an inflammatory response or a fibrotic response based on the cytokine patterns induced after exposure to the biomaterials used as soft tissue fillers. In addition, the observed alteration of cell cycle-related pathways and indication for cell cycle arrest could be an alarm response instigated by the different fillers to prevent the propagation of dysfunctional cells. Thus, the combination of evaluation of cytokine induction and effects on gene expression presents promising opportunities for the development of methods to identify potential cellular and/or molecular processes that may be predictive for biomaterial-induced responses in patients.

## 4. Materials and Methods

### 4.1. Assays for the Evaluation of Biological Effects

#### 4.1.1. Materials

The five experimental HA preparations with differences in cross-linking degree were synthesized by the National Institute for Public Health and the Environment (Rijksinsituut voor Volksgezondheid en Milieu, RIVM) and designated RIVM 1–RIVM 5. The synthesis and analysis of these experimental HA fillers was previously described in Keizers et al. [14]. HA from rooster comb was treated with BDDE according to Guarise et al. [39]. The details of this series are presented in Table 7.

Commercial fillers of the Perfectha^®^ product line (Sinclair Pharmaceuticals, Paris, France) comprise five HA dermal fillers, among which four were used. These fillers are distinguishable by their particle size, but not according to modification and cross-linking grade. For Perfectha Deep (8000 particles per mL), a uniform particle size was reported as 500 µm [40]. The Pefectha^®^ fillers contain 96% cross-linked HA and 4% HA that is not cross-linked, according to the device description of the manufacturer (Laboratoire ObvieLine, Sinclair, Dardilly, France). A more detailed product description of these fillers is shown in Table 8. Each filler was provided in a concentration of 20 mg/mL. Bio-Alcamid^®^, a non-resorbable dermal filler consisting of a three percent polyalkylimide suspension in water, was provided by Dr. Tom Decates (Erasmus MC, Rotterdam, The Netherlands) and was used as a filler with well-known long-term adverse effects. In addition, a second non-resorbable filler Aquamid^®^, a hydrogel consisting of 97.5% water and 2.5% cross-linked polyacrylamide (Contura International Ltd., London, UK), was used in some experiments.

#### 4.1.2. Sample Preparation

Hydrophilic extracts were prepared from the experimental RIVM 1–5 preparations and Perfectha^®^ HA-based filler materials according to ISO 10993-12 [41] by incubating 0.2 g/mL using tissue culture medium in the presence of 10% fetal bovine serum (FBS) (incubation for 72 ± 2 h under rotation at 37 ± 1 °C). The 72-h extraction period was applied, although it is noted in the standard that, for cytotoxicity testing, 24 h of extraction could be accepted. The authors were, however, aware of ongoing discussions in the ISO committee responsible for the standard related to this aspect. In the recent revision of the standard [42], these discussions resulted in a clear recommendation to apply a 72-h extraction period for the cytotoxicity testing of medical devices which are in prolonged (>24 h–30 d) or long-term contact (>30 d) with a patient, because extraction for 24 h may not be sufficient to obtain an extract that represents the chemicals released beyond 24 h of device use. After 72 h of extraction, the extracts were filtered with 0.22 μm filters (Corning Inc., New York, NY, USA, Cat No 8160) by centrifugation at 16,000× *g* for 20 min and evaluated for biocompatibility in a cytotoxicity assay.

#### 4.1.3. Cell Culture and Cytotoxicity Assay

The cytotoxicity assay was performed according to international standard ISO 10993-5 [43], describing cytotoxicity assays with hydrophilic extracts (e.g., tissue culture medium). Cytotoxicity was evaluated using the L929 murine fibroblast as indicated in ISO 10993-5 [40] and previously reported for the cytotoxicity evaluation of HA fillers [40,44]. RAW264.7 murine macrophages and L929 murine fibroblasts were cultured in Dulbecco’s Modified Eagle Medium/Nutrient Mixture F-12 (DMEM/F12, without phenol red, Gibco/Thermo Fisher Scientific, Bleiswijk, The Netherlands) supplemented with 10% fetal bovine serum (FBS, Greiner Bio-One, Alphen aan de Rijn, The Netherlands), 1% sodium pyruvate (Gibco), and 100 U/mL penicillin–100 μg/mL streptomycin (Gibco). Cells in the exponential growth phase were isolated, counted, and seeded in flat-bottom 96-well cell tissue culture plates at 2 × 10^4^ cells per well for RAW264.7 cells and 1 × 10^4^ cells per well for L929 cells in 100 µL cell culture medium. After 20–24 h, the cells formed a semi-confluent monolayer, tissue culture medium was removed, and 100 μL of filtered extract was added and incubated for 24 h. Then, 65 μL extract was removed, cells were carefully rinsed with 180 μL DPBS (Gibco Cat No 14190) and effects on the cells were determined.

The cytotoxic activity of the HA fillers was indirectly determined by measuring the cell viability as indicated by mitochondrial activity and membrane leakage as described below. Cell cultures of M0 macrophages derived from a human monocytic cell line were incubated with the filtered extract for 24 h. The human acute monocytic leukemia cell line (THP-1) was obtained from the American Type Culture Collection (ATCC, Rockville, USA). The cells were cultured in RPMI medium supplemented with 10% FBS, 100 U/mL penicillin and 100 µg/mL streptomycin, designated complete culture medium. THP-1 cells were differentiated into M0 macrophages using phorbol myristate acetate (PMA). THP-1 cells were seeded at a density of 0.5 × 10^6^ cells/mL in the presence of 100 ng/mL PMA for 3 h. After incubation with PMA, the medium was replaced by fresh complete culture medium for 24 h (100 µL per well for a 96-well tissue culture plates). At 24 h, the complete culture medium was removed and 100 μL of filtered extract was added and incubated for 24 h. Then, 65 μL extract was pipetted off, cells were carefully rinsed with 180 μL Dulbecco’s PBS (Gibco), and the effects on the cells were determined. Cell culture medium and supplements were purchased from Life Technologies (Thermo Fisher Scientific, Bleiswijk, The Netherlands). The M0 macrophages were incubated at 37 °C in a humidified atmosphere containing 5% CO_2_.

Cell viability was determined by using a combined Alamar Blue/CFDA-AM (AB/CFDA) assay (Alamar Blue, Roche DAL, CFDA-AM, Molecular Probes). The AB/CFDA protocol was adapted from Heusinkveld et al. [45] and Lammel et al. [46], to simultaneously determine both mitochondrial activity and membrane integrity. The mitochondrial activity of the cells was recorded as an indication of cell viability with the AB assay, which is based on the ability of the cells to reduce resazurin to the bright red fluorescent resorufin. In the same experiment, membrane integrity was indirectly assessed using a CFDA-AM assay, which is based on nonspecific cytoplasmic-esterase activity transforming the CFDA-AM into a fluorescent product. A reduced presence of fluorescence in the cells indicates dead or damaged cells by the membrane leakage of the unhydrolyzed CFDA-AM substrate and the fluorescent product. Briefly, the cells were incubated with a ten-fold dilution of Alamar Blue and 4 μM CFDA-AM in DMEM/F12. Resorufin was spectrophotometrically measured at 530/590 nm after 3 h, while hydrolyzed CF (carboxy fluorescein) was spectrophotometrically measured at 485/535 nm after 1 h. Both measurements were performed in a SpectraMax M2 spectrophotometer (Molecular Devices, LCC, San Jose, CA, USA).

The viability assay was performed in four-fold in which cell cultures were incubated with undiluted extract. Complete culture medium control was included. As positive control, DMSO in complete culture medium was used. Cell survival of treated cells was expressed relative to the viability of non-treated (medium only) control cells. Results are expressed as mean and standard deviation (SD) of three independent experiments performed on three different days. In each experiment, incubations were performed in four-fold. Significance was determined according to the Student’s *t*-test.

After the incubation with the filler extracts, the supernatant was collected and stored (−80 °C) for the evaluation of cytokine production.

#### 4.1.4. Cytokine Determination

Grotenhuis et al. [31] have suggested to establish the ratio of M1 to M2 markers as a means to assess the pro-inflammatory versus pro-fibrotic response to biomaterials. For this purpose, IL-1β and CCL18 were evaluated as M1 and M2 markers, respectively. The supernatants were analyzed by ELISA, (IL-1β ELISA, EBioscience; CCL18 ELISA, Thermo Fisher Scientific Inc., Bleiswijk, The Netherlands). The ELISA’s were performed according to the manufacturer’s instructions. Results are expressed as the mean and standard deviation (SD) of three independent experiments performed on three different days. In each experiment, incubations were performed in four-fold. Significance was determined according to the Student’s *t*-test.

### 4.2. Genome-Wide Evaluation of Effects on Gene Expression

#### 4.2.1. Macrophages Derived from THP-1 Cells

For the determination of gene expression, M0 macrophages (originating from THP-1 cells, see above) were exposed to the filler extracts for 24 h at 37 °C.

#### 4.2.2. RNA Isolation

After exposure cells were harvested and stored in 1 mL QIAzol, the total RNA was isolated using the miRNeasy isolation kit (Qiagen, Venlo, The Netherlands) as instructed by the manufacturer. After isolation, RNA concentrations were measured on a NanoDrop spectrophotometer and the integrity was determined with a Bioanalyzer 2100 (Agilent Technologies, Amstelveen, The Netherlands). Only samples with a RNA integrity number (RIN) > 6 were used for hybridization. An average RIN > 9 was measured. The extracted RNA was stored at −80 °C until it was used as a template for cDNA synthesis.

#### 4.2.3. Microarray Analysis

From each sample, 0.2 μg total RNA was used to synthesize fluorescent cyanine-3-labeled cRNA following the Agilent one-color Quick-Amp labeling protocol (Agilent Technologies, Amstelveen, The Netherlands). Then, the samples were hybridized on Agilent SurePrint G3 Human GE v2 8 × 60 K Microarrays according to the manufacturer’s instructions. After washing the microarrays, they were scanned using the Agilent G2505C DNA Microarray Scanner (Agilent Technologies). Raw data on the pixel intensities were extracted from the scan images using Agilent’s Feature Extraction Software. The extracted data were checked for its quality using ArrayQC [47], an in-house developed quality control (QC) pipeline in R. For each spot, the following steps were taken: local background correction, flagging of bad spots, controls, and spots with excessively low intensity, log2 transformation, and quantile normalization. Further preprocessing included the omission of flagged probes and merging of replicate probes based on median.

Overall, out of the performed microarrays, two did not pass the QC cut-off criteria and therefore, were omitted from further analyses.

Pre-processing of the data as described above was performed independently for two sets of experiments:

- M0 macrophages derived from THP-1 cells exposed to RIVM preparations and Bio-Alcamid^®^;
- M0 macrophages derived from THP-1 cells exposed to Perfectha^®^ fillers.

#### 4.2.4. Gene Expression Analysis

Differentially expressed genes (DEGs) resulting from the exposure of the THP-1-derived M0 macrophages to the different dermal fillers were identified. For each of the selected groups of the in vitro experiments, a paired analysis between the exposures and their controls was conducted using a moderated *t*-test of the Limma (Linear Models for Microarray Data) R-package. Cut-off criteria used with this test to define significance was a Benjamini–Hochberg adjusted *p*-value < 0.05. In addition, the number of genes with an absolute fold change (FC) > 1.5 (corresponds with a log2 fold change of 0.585) were captured.

#### 4.2.5. Pathway Analysis

The selected DEGs for each in vitro exposure were analyzed by an over-representation analysis in PathVisio (https://www.pathvisio.org/ accessed on 23 May 2022). The statistical analysis in PathVisio provides a Z-score for each pathway. Pathways were considered significantly enriched if the Z-score was higher than 1.96, which is based on the assumptions that the data have a hypergeometric distribution: a Z-score of 1.96 agrees with a *p*-value of 0.05.

## Figures and Tables

**Table 1:** Differentially expressed genes (DEGs) for M0 macrophages derived from THP-1 cells exposed to RIVM preparations and Bio-Alcamid^®^ extracts. The number of genes significantly different compared to non-treated control cells is indicated in bold.

THP-1	Bio-Alcamid^®^	RIVM 1	RIVM 2	RIVM 3	RIVM 4	RIVM 5
|FC| ≥ 1.5	4683	941	679	999	370	395
Up-regulated	2407	791	271	511	187	284
Down-regulated	2276	150	408	488	183	111
**Adj. *p*-value < 0.05**	**10,781**	**108**	**234**	**73**	**0**	**1**
|FC| and adj. *p*-value	4454	55	153	49	0	0
**Adj. *p*-value < 0.2**	**14,835**	**1773**	**1025**	**1453**	**0**	**1**

**Table 2:** Differentially expressed genes (DEGs) for M0 macrophages derived from THP-1 cells exposed to Perfectha^®^ filler extracts. The number of genes significantly different compared to non-treated control cells is indicated in bold.

THP-1	FineLines	Derm	Deep	SubSkin
|FC| ≥ 1.5	640	607	421	485
Up-regulated	190	161	262	182
Down-regulated	450	446	159	303
**Adj. *p*-value < 0.05**	**735**	**572**	**287**	**227**
|FC| and adj. *p*-value	246	215	99	93
**Adj. *p*-value < 0.2**	**2096**	**2988**	**1550**	**3183**

**Table 3:** Gene expression changes of *IL-1β* and *CCL18* from M0 macrophages derived from THP-1 cells exposed to RIVM preparations, Bio-Alcamid^®^ and Perfectha^®^ filler extracts. Significant gene expression with adjusted *p*-value < 0.05 and adjusted *p*-value < 0.2 are indicated in bold and in italics, respectively.

	*IL-1β*		*CCL18*	
	Log2 Fold Change	Adjusted *p*-Value	Log2 Fold Change	Adjusted *p*-Value
RIVM 1	0.09108	0.842737	1.255873	0.210881
RIVM 2	0.073431	0.884412	*1.33759*	*0.161463*
RIVM 3	0.001202	0.998126	1.03912	0.312679
RIVM 4	0.064341	0.959091	0.688105	0.731468
RIVM 5	0.08153	0.933837	0.606583	0.80213
SubSkin	0.008054	0.986642	*0.631015*	*0.178301*
Deep	−0.05681	0.866025	*0.908939*	*0.052843*
Derm	0.006694	0.986972	*0.730659*	*0.083834*
FineLines	0.048485	0.87731	*0.622876*	*0.128307*
Bio-Alcamid	**1.80336**	**9.97 × 10^−8^**	**5.43877**	**1.24 × 10^−7^**

**Table 4:** Top 10 significant pathways for M0 macrophages derived from THP-1 cells exposed to Bio-Alcamid^®^ extract. DEGs selected by moderated *t*-test with adjusted *p*-value < 0.05 were used for pathway analysis in PathVisio. Pathways related to immune and inflammatory responses are shown in bold.

Pathway	Z-Score	*p*-Value (Permuted)
**IL-3 signaling pathway**	2.7	0.009
Apoptosis modulation by HSP70	2.52	0.002
**Macrophage markers**	2.49	0.005
Mevalonate arm of cholesterol biosynthesis pathway	2.47	0.013
**Kit receptor signaling pathway**	2.44	0.01
**IL-4 signaling pathway**	2.42	0.013
**Interferon type I signaling pathways**	2.42	0.017
**Signal transduction through IL1R**	2.25	0.025
Splicing factor NOVA regulated synaptic proteins	2.12	0.035
p53 transcriptional gene network	2.11	0.029

**Table 5:** Top 5 significant pathways for M0 macrophages derived from THP-1 cells exposed to RIVM 1, 2, and 3 preparations extracts. DEGs selected by moderated *t*-test with adjusted *p*-value < 0.2 were used for pathway analysis in PathVisio. Pathways related to cell cycle control are shown in bold.

Pathway	Z-Score	*p*-Value (Permuted)
**RIVM 1**		
**G1 to S cell cycle control**	4.96	0
**DNA replication**	4.37	0
MFAP5-mediated ovarian cancer cell motility and invasiveness	3.76	0.003
Hepatitis C and hepatocellular carcinoma	3.61	0.001
**Retinoblastoma gene in cancer**	3.47	0.001
**RIVM 2**		
**Retinoblastoma gene in cancer**	14.24	0
**DNA replication**	10.78	0
**G1 to S cell cycle control**	7.86	0
**Cell cycle**	6.27	0
**DNA mismatch repair**	4.92	0
**RIVM 3**		
**Retinoblastoma gene in cancer**	12.48	0
**DNA replication**	9.5	0
**G1 to S cell cycle control**	7.69	0
**Cell cycle**	5.98	0
Vitamin B12 disorders	4.92	0

**Table 6:** Top 5 significant pathways for M0 macrophages derived from THP-1 cells exposed to Perfectha^®^ filler extracts. DEGs selected by moderated *t*-test with adjusted *p*-value < 0.05 were used for pathway analysis in PathVisio. Pathways related to cell cycle control and those related to immune and inflammatory responses are shown in bold and in italics, respectively.

Pathway	Z-Score	*p*-Value (Permuted)
**FineLines**		
**DNA replication**	8.66	0
**Retinoblastoma gene in cancer**	8.47	0
**G1 to S cell cycle control**	7.85	0
Hepatitis C and hepatocellular carcinoma	5.08	0
**Cell cycle**	5.07	0
**Deep**		
Pentose phosphate metabolism	7.98	0
ID signaling pathway	6.62	0
Ultraconserved region 339 modulation of tumor suppressor microRNAs in cancer	6.06	0
*Antiviral and anti-inflammatory effects of Nrf2 on SARS-CoV-2 pathway*	5.7	0
Oxidative stress response	5.4	0
**Derm**		
**DNA replication**	9.01	0
**Retinoblastoma gene in cancer**	8.66	0
**G1 to S cell cycle control**	8.35	0
Pentose phosphate metabolism	5.55	0
**Cell cycle**	5.15	0
**SubSkin**		
**Retinoblastoma gene in cancer**	4.96	0
**G1 to S cell cycle control**	4.37	0
Ultraconserved region 339 modulation of tumor suppressor microRNAs in cancer	3.76	0.003
*Antiviral and anti-inflammatory effects of Nrf2 on SARS-CoV-2 pathway*	3.61	0.001
**miRNAs involved in DNA damage response**	3.47	0.001

**Table 7:** Experimental synthesized cross-linked HA used in the biocompatibility assays. Modification and cross-linking grade were determined by LC-MS.

Product	Amount HA (mg)	Amount BDDE (µL)	Volume (mL)	Modification Grade (%)	Cross-Linking Grade (%)
RIVM 1	496.6	60.0	25	10.7	1.9
RIVM 2	496.5	113.6	25	22.2	4.5
RIVM 3	500.7	170.5	25	31.5	7.6
RIVM 4	500.0	227.3	25	36.6	9.9
RIVM 5	500.5	454.5	25	42.9	14.4

**Table 8:** Product description of Perfectha^®^ Hyaluronic Acid dermal fillers.

	FineLines	Derm	Deep	SubSkin
Indication for use	Fine lines and superfacial wrinkles	Moderate correction to face and lip contour	Deep wrinkles and furrows and lip augmentation	Volume augmentation cheeks, chin, jaw line
Injection method	Intradermal	Subcutaneous	Subcutaneous	Deep subcutaneous to supraperiosteal
Volume	0.5 mL	1 mL	1 mL	3 mL
No of particles (Gel/mL)	180,000	90,000	8000	2000
Duration of effect	4–6 months	6–12 months	8–12 months	12–18 months
Needles used	2 × 30 G 1/2	2 × 30 G 1/2	2 × 27 G 1/2	22 G and cannula

## Data Availability

The data presented in this study are available in the article and the Supplementary Material.

## Supplementary Materials

The following supporting information can be downloaded at: https://www.mdpi.com/article/10.3390/ijms23137275/s1.
