# Supplementary material for: Evaluation of Adverse Effects of Resorbable Hyaluronic Acid Fillers: Determination of Macrophage Responses
Source: Int J Mol Sci. 2022 Jun 30;23(13):7275. doi: 10.3390/ijms23137275 (PMC9266407; doi:10.3390/ijms23137275)
Supplement: Supplementary file 1 [file ijms-23-07275-s001.zip › De Jong et al IJMS Supplementary Materials.pdf]

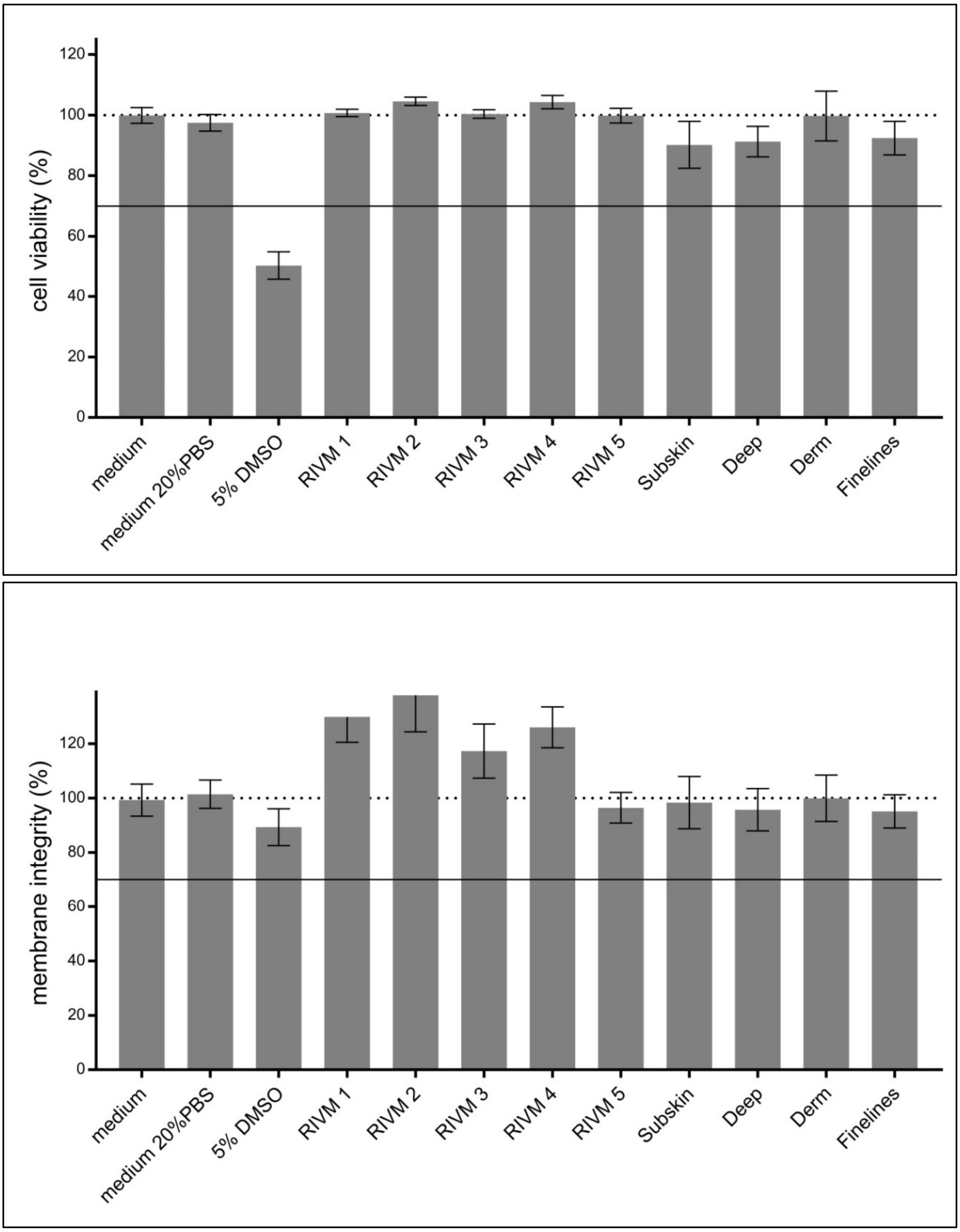

Supplementary Figure S1. Effect of RIVM and Perfectha® (Subskin, Deep, Derm, Finelines) HA fillers, and DMSO on cell viability of L929 cells after incubation with filler extracts or DMSO for 24 hours. Results are presented as mean ± SD of three independent experiments. Top metabolic activity of the cells as indicated by Alamar Blue conversion, bottom membrane integrity as indicated by the conversion of CFDA-AM substrate in carboxy fluorescein retained in the cells. The horizontal line indicates a cellular activity of 70% compared to non-treated control cells, below which a toxic effect is indicated (ISO 10993-5). RIVM fillers #1 to #5 show increased cross linking of hyaluronic acid chains by BDDE. Perfectha® fillers show decrease in particle size from Perfectha®Subskin to Perfectha® Finelines.

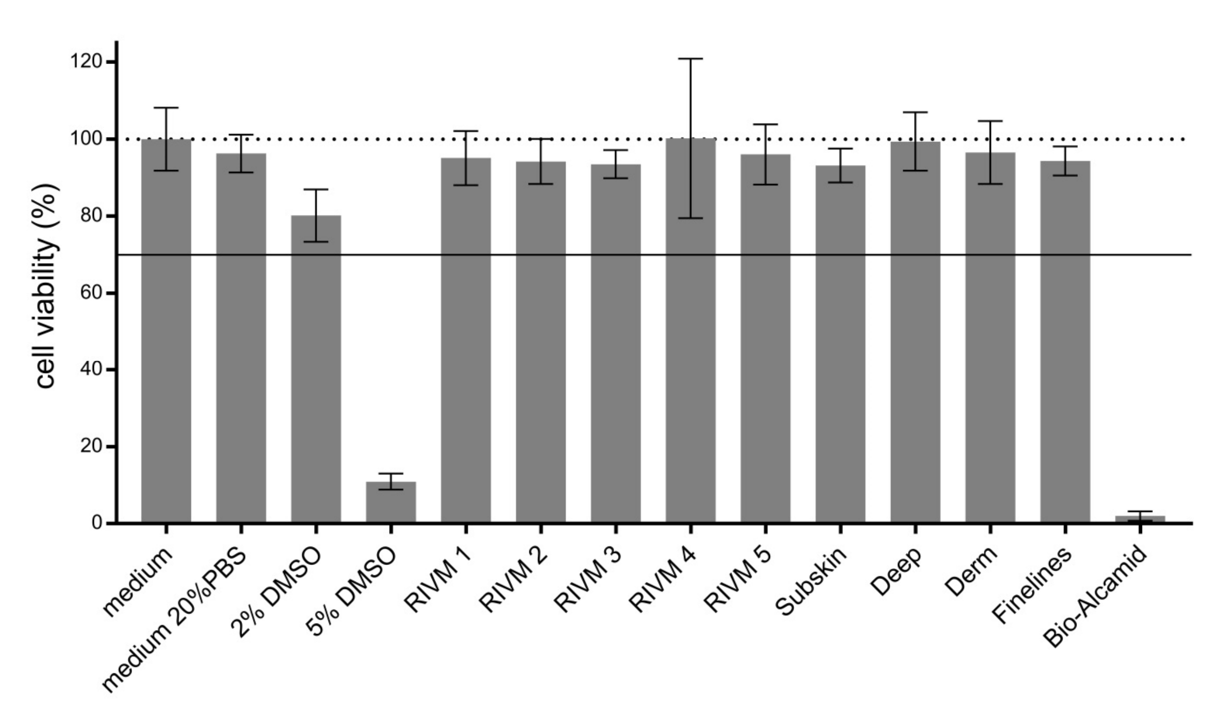

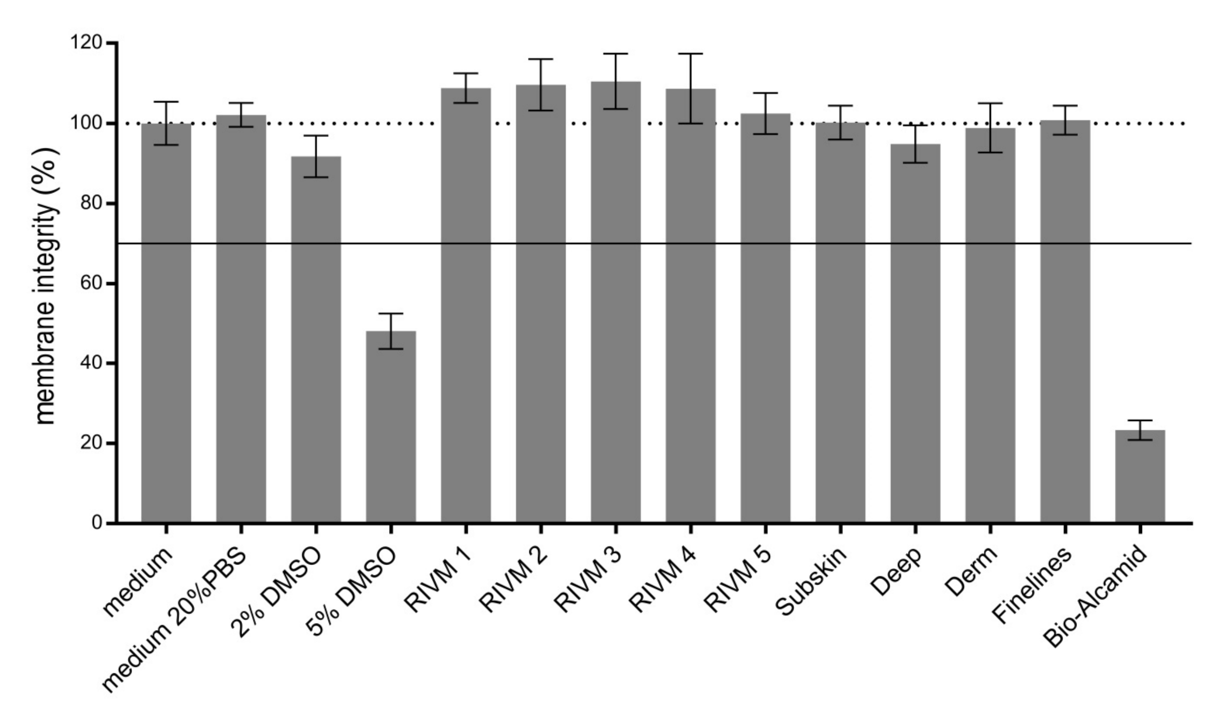

Supplementary Figure S2. Effect of RIVM and Perfectha (Subskin, Deep, Derm, Finelines) HA fillers, DMSO and non resorbable BioAlcamid HA filler on RAW264.7 macrophages after incubation with filler extracts or DMSO for 24 hours. Results are presented as mean ± SD of three independent experiments. Top metabolic activity of the cells as indicated by Alamar Blue conversion. Bottom membrane integrity as indicated by the conversion of CFDA-AM substrate in carboxy fluorescein . The horizontal line indicates a cellular activity of 70% compared to non-treated control cells, below which a toxic effect is indicated (ISO 10993-5). RIVM fillers #1 to #5 show increased cross linking of hyaluronic acid chains by BDDE. Perfectha® fillers show decrease in particle size from Perfectha®Subskin to Perfectha® Finelines.

Supplementary Table S1. Output of the gene expression analyses for the cytokines and chemokines. For each of the selected groups of the in vitro experiments a paired analysis between the exposures and their controls was conducted using a moderated t-test of the Limma (Linear Models for Microarray Data) R-package. Log2 fold changes, p-values and Benjamini-Hochberg adjusted p-values are provided. Adjusted p-values <0.2 are high-lighted in red. Rows containing at least one adjusted p-value <0.2 are in bold.

De Jong et al IJMS
Suppl Table S1.xlsx

Supplementary Table S2. Over-representation analysis in PathVisio of differentially expressed genes (adjusted pvalue <0.05 or <0.2) from THP-1 derived macrophages exposed to Bio-Alcamid®, RIVM preparations and Perfectha® fillers.

De Jong et al IJMS
Suppl Table S2.xlsx

Supplementary Table S3. Output of the gene expression analyses for genes of the cell cycle (pathway). For each of the selected groups of the in vitro experiments a paired analysis between the exposures and their controls was conducted using a moderated t-test of the Limma (Linear Models for Microarray Data) R-package. Log2 fold changes, p-values and Benjamini-Hochberg adjusted p-values are provided. Adjusted p-values <0.2 are high-lighted in red. Rows containing at least one adjusted p-value <0.2 are in bold.

De Jong et al IJMS
Suppl Table S3.xlsx
